# Efficacy of interceptor® G2, a long-lasting insecticide mixture net treated with chlorfenapyr and alpha-cypermethrin against *Anopheles funestus*: experimental hut trials in north-eastern Tanzania

**DOI:** 10.1186/s12936-021-03716-z

**Published:** 2021-04-09

**Authors:** Patrick K. Tungu, Elisante Michael, Wema Sudi, William W. Kisinza, Mark Rowland

**Affiliations:** 1 Amani Medical Research Centre, National Institute for Medical Research, Muheza, Tanzania; 2 Pan-African Malaria Vector Research Consortium (PAMVERC), P.O.Box 81, Muheza, Tanga, Tanzania; 3 London School of Hygiene and Tropical Medicine, London, UK

**Keywords:** Long-lasting insecticidal nets, Interceptor G2, Chlorfenapyr, Insecticide resistance, *Anopheles funestus*, Experimental huts, Tanzania

## Abstract

**Background:**

The effectiveness of long-lasting insecticidal nets (LLIN), the primary method for preventing malaria in Africa, is compromised by evolution and spread of pyrethroid resistance. Further gains require new insecticides with novel modes of action. Chlorfenapyr is a pyrrole insecticide that disrupts mitochrondrial function and confers no cross-resistance to neurotoxic insecticides. Interceptor® G2 LN (IG2) is an insecticide-mixture LLIN, which combines wash-resistant formulations of chlorfenapyr and the pyrethroid alpha-cypermethrin. The objective was to determine IG2 efficacy under controlled household-like conditions for personal protection and control of wild, pyrethroid-resistant *Anopheles funestus* mosquitoes.

**Methods:**

Experimental hut trials tested IG2 efficacy against two positive controls—a chlorfenapyr-treated net and a standard alpha-cypermethrin LLIN, Interceptor LN (IG1)—consistent with World Health Organization (WHO) evaluation guidelines. Mosquito mortality, blood-feeding inhibition, personal protection, repellency and insecticide-induced exiting were recorded after zero and 20 washing cycles. The trial was repeated and analysed using multivariate and meta-analysis.

**Results:**

In the two trials held in NE Tanzania, *An. funestus* mortality was 2.27 (risk ratio 95% CI 1.13–4.56) times greater with unwashed Interceptor G2 than with unwashed Interceptor LN (p = 0.012). There was no significant loss in mortality with IG2 between 0 and 20 washes (1.04, 95% CI 0.83–1.30, p = 0.73). Comparison with chlorfenapyr treated net indicated that most mortality was induced by the chlorfenapyr component of IG2 (0.96, CI 0.74–1.23), while comparison with Interceptor LN indicated blood-feeding was inhibited by the pyrethroid component of IG2 (IG2: 0.70, CI 0.44–1.11 vs IG1: 0.61, CI 0.39–0.97). Both insecticide components contributed to exiting from the huts but the contributions were heterogeneous between trials (heterogeneity Q = 36, P = 0.02). WHO susceptibility tests with pyrethroid papers recorded 44% survival in *An. funestus*.

**Conclusions:**

The high mortality recorded by IG2 against pyrethroid-resistant *An. funestus* provides first field evidence of high efficacy against this primary, anthropophilic, malaria vector.

## Background

Long-lasting insecticidal nets (LLINs) are essential for malaria transmission control in sub-Saharan Africa [1]. The halving of the malaria burden over the last 15 years is largely attributed to increasing coverage of pyrethroid LLIN, which culminated in universal free distribution across all age groups in Africa [2]. Concurrent with this public health achievement and cultural shift in sleeping behaviour has been the evolution and spread of pyrethroid resistance across Africa in the two primary vector mosquito species complexes.

Pyrethroids, owing to their efficacy, safety and low-cost were once the only insecticides approved for use on LLINs [3]. Since 2015, further reduction in the annual malaria burden has stalled, and pyrethroids are no longer deemed sufficient on their own [1]. The evolution of severe resistance was anticipated, and when the first signs of field failure were reported in 2007 [4], steps had already been taken to identify alternatives [5, 6]. The first active ingredient (AI) to be developed by pesticide industry to enhance pyrethroid efficacy on nets was the synergist PBO [7, 8]. This supplemental compound, long used in domestic fly sprays to enhance pyrethroid toxicity, can neutralize metabolic mechanisms responsible for resistance to pyrethroids. Several brands of pyrethroid-PBO LLIN are currently being scaled up in countries where monooxygenase resistance mechanisms are contributing to impairment or loss of malaria control [9–11]. Pyrethroid-PBO LLIN is no panacea; it cannot neutralize all pyrethroid resistance mechanisms that have evolved, and this is no time for complacency. What is needed is an array of alternative insecticides that can complement the pyrethroids on Dual-AI LLIN. This is no trivial task as alternative insecticides for nets need to be safe to humans, toxic to mosquitoes, wash-tolerant on nets and exhibit no cross resistance to pyrethroids. One such insecticide, which is showing promise, is the pyrrole chlorfenapyr [12]. After 15 years of development and evaluation in laboratory and small-scale experimental hut trials against anopheline mosquitoes [13–18], the first cluster randomized trials (CRT) of a LLIN that combines chlorfenapyr with pyrethroid in a wash-tolerant formulation are currently underway and are due to report in 2021 in Tanzania, East Africa and in 2022 in Benin, West Africa. Epidemiological evidence of effectiveness against malaria in CRT is a prerequisite before the World Health Organization (WHO) will grant recommendation of any new class of LLIN for malaria control. Both CRTs are targeting the *Anopheles gambiae* complex: *An. gambiae *sensu stricto (*s.s*.) in NW Tanzania and *Anopheles coluzzii* in Benin. However, a third primary vector has re-emerged, *Anopheles funestus* [19], and this species is becoming the predominant vector along the eastern seaboard of Tanzania after a hiatus of several years when LLIN were first taken to scale in mass distribution campaigns and control of the then pyrethroid-susceptible *An. funestus* and *An. gambiae* was achieved [20, 21]. The return of both *An. funestus* and *An. gambiae s.s.* is in pyrethroid-resistant form.

The sibling species, vector competence and insecticide resistance status of the *An. funestus* complex has only recently been characterized in Tanzania [22]. Molecular identification of collections from 2005–2014 in NE Tanzania revealed *An. funestus s.s.* (97%) as the predominant species and *Anophelesrivulorum* (2%) and *Anopheles leesoni* (1%) as minor sibling species. *Plasmodium falciparum* CSP positivity was 8.3% for recently collected *An. funestus s.s.* [22]. *Wucheria bancrofti* infection rates decreased from 14.8% in the 2005–2007 archived specimens to only 0.5% in newly collected specimens, with 93% of filarial infections confined to *An. funestus s.s.* The high *P. falciparum* and decreasing *W. bancrofti* infections in *An. funestus s.s.* most likely reflects infection levels of these parasites in the human population and confirms its vectorial importance [22]. Vector surveys further south in coastal Bagamoyo and Kilombero valley produced similar trends and species ratios as NE Tanzania. In Bagamoyo, there was 84% *An. funestus s.s*., 13.6% *An. leesoni*, 1.5% *An. rivulorum*, and 0.6% *Anopheles parensis* [23]. In Kilombero valley, 97% were *An. funestus s.s.,* 2% *An. rivorulum* and 1% *An. leesoni* [24].

*Anopheles funestus s.s.* and *An. gambiae* s*.s.* in addition to being pyrethroid resistant are naturally highly anthropophagic and endophilic. These are the primary vector species to target with the new generation insecticides like chlorfenapyr. Unlike pyrethroids and other conventional public health insecticides which are neurotoxic, chlorfenapyr disrupts the oxidative pathways that enable proton transfer, conversion of ADP to ATP and cellular respiration in mitochondria [15, 25]. With its non-neurological mode of action, chlorfenapyr shows no cross resistance to insecticide classes normally used for vector control and hence is a leading candidate for targeting vector species resistant to standard neurotoxic insecticides [13, 17]. When evaluated on hand-treated mosquito nets against wild mosquitoes in experimental huts, chlorfenapyr showed improved control of mosquitoes resistant to WHO-approved insecticides [14, 26].

Interceptor G2 LN (IG2) is a Dual-AI LLIN developed by the manufacturer BASF SE which is designed to provide protection against pyrethroid-resistant mosquitoes by means of a mixture of chlorfenapyr and alpha-cypermethrin in a long-lasting wash-resistant formulation. The first experimental hut trials of IG2, undertaken in Benin, Burkina Faso and Cote d’Ivoire in West Africa, targeted members of the *An gambiae* complex: *An coluzzii*, *An gambiae s.s.* and *Anopheles arabiensis* [15, 27, 28]. The present paper reports on sequential hut trials in NE Tanzania on the East African seaboard designed to assess the efficacy of Interceptor G2 LN against the primary East African vectors *An. funestus s.s.* and *Anopheles gambiae s.s*. IG2 was tested unwashed and after 20 standardized washes as proxy for an ageing net consistent with WHO guidelines for evaluating LLIN. Two other net types served as positive controls: the pyrethroid-only Interceptor LN (IG1) and a net hand-treated with chlorfenapyr SC formulation. While it was anticipated that pyrethroid resistant *An. funestus s.s.* and *An. gambiae s.s.* would both be present, on these two occasions only *An. funestus s.s.* was present in significant densities.

## Methods

### Study site and experimental huts

Two experimental hut studies were conducted in Muheza district, Tanga region, at the field station in Zeneti (5°13′ S, 38°39′ E, 193 m altitude), where *An. gambiae s.s*. and *An. funestus s.s.* are the major malaria vectors [20, 22]. Polymerase chain reaction sibling species analysis of 500 *An. funestus* collected from Zenet between 2016–2017 results showed all were *An. funestus s.s*. In World Health Organization insecticide susceptibility tests using permethrin papers conducted on F1 adult mosquitoes from Zeneti in the year before the hut trials, mortality was 56% among *An. gambiae s.s.* and 62% among *An. funestus*. In intensity bottle bioassay *An. gambiae s.s.* showed 30-fold resistance to permethrin relative to susceptible Kisumu strain [30]. There was no resistance to carbamates or organophosphates.

The WHO Phase II evaluation of Interceptor G2 was conducted in 6 experimental huts of the East African design [31]. The operating principle of the huts is described in WHO LLIN evaluation guidelines [32]. The hut design allows host-seeking mosquitoes unfettered access though two open eave gaps, 5 cm deep and 3 m wide, between wall and roof on two sides of the hut, attracted by the human host sleeping inside, and captures surviving mosquitoes exiting into window traps fitted on two of the walls or into verandah traps accessed through eave gaps above the walls. Other features include a ceiling lined with hessian sackcloth similar to thatch, a corrugated iron roof, a concrete plinth and water-filled moat to deny entry to scavenging ants. The eaves of the two unscreened verandahs were baffled inwardly to funnel host-seeking mosquitoes into the hut and to deter exiting through the same eave gaps. Two screened and closed veranda traps located on the other two sides of the hut, and two baffled window traps, capture any mosquito that exit the rooms via the two open eaves or windows. With this modification to the traditional verandah hut design there was no need to make any correction for escaping mosquitoes because all escapees are recorded [31].

### Experimental hut trial design

Two experimental huts trials were undertaken. The first trial was conducted over 54 collection nights between November and December 2015, the second trial was conducted for 36 nights between May and July 2016. The following six treatment arms were included:

(i) Untreated polyester net,
(ii) Interceptor LN, unwashed
(iii) Interceptor LN, washed 20 times
(iv) Interceptor G2 LN, unwashed
(v) Interceptor G2 LN, washed 20 times

Polyester net, conventionally treated with chlorfenapyr SC formulation (Phantom 21% SC, BASF) at 200 mg/m^2^.

Washing of LLINs was done according to WHO Phase II protocols [32]. The interval between washes was 1 day which is the established regeneration time for Interceptor G2 and Interceptor LN [8]. Each net was cut with six holes of 4 cm diameter to simulate wear and tear. For the washed nets, washing was done in 10 L of soap solution (2 g/l of Savon de Marseille). Nets were agitated for 3 min by stirring with a pole, then allowed to soak for four minutes, and then stirred again for 3 min. The nets were rinsed twice using the same procedure with clean tap water. All nets were 100-denier. Three nets were used per treatment arm.

Treatments were rotated between huts each week (3 nets tested 3 times over 9 days or 2 times over a 6-days) with sleepers rotated between huts and treatments each night using a randomized latin square design to adjust for variation in personal attractiveness to mosquitoes or hut positional effect. Each morning mosquitoes were collected and held for 72 h in cups with sugar solution to record any delayed mortality. All dead and surviving mosquitoes were retained on silica gel for molecular identification [33] and for genotyping of L1014S or L1014F *kdr* alleles using Taqman PCR [34].

The outcomes of the hut trials were:

(i) Deterrence—the proportional reduction of mosquito entry into huts with insecticide treated nets relative to huts with untreated nets
(ii) Mortality—the proportion of mosquitoes killed by a treatment relative to the total numbers entering huts with that treatment
(iii) Overall killing effect—the number of mosquitoes killed by a treatment relative to the number dying in the untreated control, as derived from the formula: *killing effect (%)* = *100 (Kt-Ku)/Tu,* where i) Kt is the number killed in the huts with treated nets, ii) Ku is the number dying in the huts with untreated nets, iii) Tu is the total entering the huts with untreated nets
(iv) Blood-feeding inhibition—the proportional reduction blood-feeding in huts with treated nets relative to the proportion blood-feeding in huts with untreated nets
(v) Personal protection—the reduction in the numbers of mosquitoes blood-feeding in huts with treated nets relative to numbers blood-feeding through untreated nets, as derived from the formula: *% Personal protection* = *100 (Bu-Bt)/Bu,* where (i) Bu is the total number of blood-fed mosquitoes in the huts with untreated nets, and (ii) Bt is the total number of blood-fed mosquitoes in huts with treated nets
(vi) Insecticide induced exiting–the proportional increase in exiting from huts with insecticide treated nets relative to the proportion exiting from huts with untreated nets

### Chemical analysis

Netting samples were cut from each net before and after washing and after completion of the trial for determination of insecticide content. Determination of alpha-cypermethrin and chlorfenapyr content was performed at BASF (1st trial) and Walloon Agricultural Research Centre (CRA-W) (2nd trial) using a draft CIPAC method jointly developed by CRA-W and BASF based on CIPAC 454/LN/M/3.1. The method involves extraction of alpha-cypermethrin and chlorfenapyr by ultrasonication at ambient temperature for 30 min in heptane in the presence of dicyclohexyl phthalate as internal standard, by adding citric acid, and determination by gas chromatography with flame ionization detection (GCFID). The insecticide concentration of each sample (g/kg) was converted to mg/m^2^ before presentation.

### Supplementary bioassay tests on nets used in the trials

#### Mosquito strains

*Anopheles gambiae s.s.* Kisumu, a laboratory insecticide susceptible strain, originally from Kenya.

*Anopheles gambiae s.s.* Zeneti, a pyrethroid resistant strain of *An. gambiae s.s.* from Zeneti village containing the L1014S pyrethroid resistance knockdown allele (*kdr* east) [29] and showing 30-fold resistance to permethrin relative to susceptible *An. gambiae* Kisumu.

#### WHO cone bioassays

These were conducted on standardised washed and unwashed nets to estimate the wash fastness of each net formulation. Five pieces were cut from each net and two replicates of five susceptible or resistant *An. gambiae* mosquitoes were exposed for 3 min. Mortality was scored at 24 h, 48 h and 72 h post-exposure.

#### Tunnel tests

These were conducted on standardised washed and unwashed pieces of Interceptor G2 LN netting after 0 and 20 washes. A total of 100 susceptible and resistant mosquitoes were tested in tunnel tests in replicates of 50 mosquitoes per test in accordance with WHO guidelines [32]. The tunnels were divided into two sections by a netting frame punctured with 9 holes slotted across the tunnel. In one section an anaesthetized guinea pig was housed unconstrained in a cage to attract mosquitoes from the release section overnight. Test conditions were 25 ± 2 °C and 80 ± 10% RH. Mosquito mortality was recorded after 24 h and 72 h holding periods.

### Statistical analysis

Data were entered into an Excel database and transferred to Stata 11 (Stata Corp LP, College Station, TX, USA) for processing and analysis. Cone bioassays and tunnel test data were analysed using logistic regression for grouped data adjusting for clustering within replicate tests.

Proportional outcomes in the experimental hut trial (mortality, blood-feeding, exiting) related to each treatment were assessed using logistic regression for grouped data adjusting for daily collected mosquitoes. In addition to the fixed effect of each treatment, each model included random effects to account for variation between the hut position and sleeper attractiveness. Comparison between numeric outcomes of treatments (personal protection, killing effect, deterrence) was analysed using negative binomial regression with adjustment for variation in the same covariates described above.

Risk ratios of mortality, blood-feeding and exiting rates the two trials were pooled using meta-analysis using a random-effects model STATA® statistical analysis software package version 16 (Stata corporation, Collage Station, Texas 77,845 USA, 2019). Overall heterogeneity across trials was calculated using Cochrane’s Q test with a P value of less than 0.05 to indicate statistical heterogeneity and quantified heterogeneity using the I^2^ statistic [35, 36].

## Results

### Resistance status

WHO susceptibility tests using permethrin and alpha-cypermethrin treated papers were conducted against F1 progeny of mosquitoes collected from huts containing untreated nets before and during the trial. Mortality recorded using 0.75% permethrin papers was 46.7% for *An. gambiae* and 56.7% for *An. funestus* in the first trial (2015) and 43% and 52.6%, respectively in the second (2016), indicating resistance to pyrethroids in both species. Mortality using 0.05% alpha-cypermethrin papers was 52.7% for *An. gambiae* during the first trial. Alpha-cypermethrin papers were not available during the 2^nd^ trial, but other alpha-cyano pyrethroids such as 0.05% deltamethrin and 0.05% lambdacyhalothrin gave a similar 73.8% and 50.6% mortality respectively. Concurrent mortality using the same insecticide test papers against susceptible *An. gambiae* Kisumu was 100% in each case. Insecticide resistance intensity testing showed Zenet field *An. gambiae* to have over 30-fold resistance to pyrethroid (permethrin) compared to susceptible *An. gambiae* Kisumu.

## Phase II—experimental hut trials

### Mosquito entry into experimental huts

The average number of mosquitoes entering and exiting the hut are shown in Table 1. The geometric mean number of *An. funestus* collected during the first trial ranged from 0.6 to 1.5 per hut per night. During the second trial the geometric mean number of *An. funestus* ranged from 1.3 to 1.8 per hut per night. In both trials significantly fewer *An. funestus* were collected from the huts with chlorfenapyr CTN compared to the huts with the untreated nets. No consistent deterrent effect was observed with IG1 (alpha-cypermethin alone) or IG2 compared to untreated nets.

**Table 1:** *Anopheles funestus* mosquitoes collected and exiting into verandah and window traps during the two experimental hut trials

Trial	Effect	Untreated net	Interceptor LN	Interceptor LN	Interceptor G2	Interceptor G2	Chlorfenapyr CTN
		0 washes	0 washes	20 washes	0 washes	20 washes	0 washes
1	Total number caught (mean/night)	139 (2.6)	94 (1.7)	97 (1.8)	63 (1.2)	133 (2.5)	54 (1.0)
	Geometric mean per night (CI)	1.4^a^ (0.9–1.9)	1.1^ab^ (0.7–1.5)	1.3^a^ (0.9–1.7)	0.9^ab^ (0.6–1.2)	1.5^a^ (1.0–2.1)	0.6^b^ (0.4–0.9)
	% Deterrence	0	32.4	30.2	54.7	4.3	61.2
2	Total number caught (mean/night)	111 (3.1)	80 (2.2)	78 (2.2)	103 (2.9)	86 (2.4)	74 (2.1)
	Geometric mean per night (CI)	1.8^a^ (1.1–2.8)	1.5^b^ (1.0–2.2)	1.8^ab^ (1.2–2.4)	1.6^ab^ (0.9–2.4)	1.3^b^ (0.8–2.0)	1.3^b^ (0.8–2.0)
	% Deterrence	0	27.9	29.7	7.2	22.5	33.3
1	Total in verandah & window traps	59	74	83	51	107	32
	% Exiting (95% C.I.)	42^a^ (27–59)	79^c^ (67–87)	86^c^ (73–93)	81^c^ (68–90)	80^c^ (69–89)	59^b^ (39–77)
2	Total in verandah & window traps	75	53	51	48	46	58
	% Exiting (95% C.I.)	68^ac^ (52–80)	66^ac^ (51–79)	65^c^ (54–75)	47^b^ (31–63)	54^bc^ (36–70)	78^a^ (67–87)

The numbers in the same row sharing the same letter superscript do not differ significantly (p > 0.05)

### Mortality and overall killing effect

**Fig. 1:**
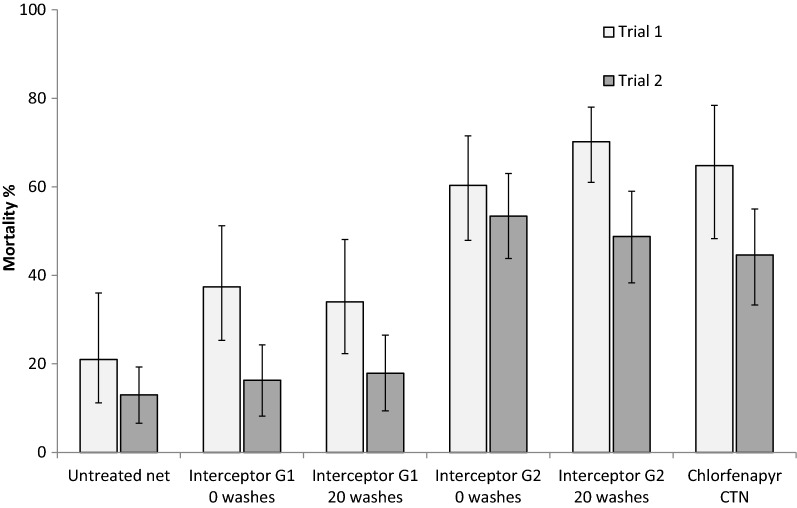
Experimental hut trials of Interceptor G2 and Interceptor LN in NE Tanzania: mortality of free-flying *Anopheles funestus* after 72 h holding period

The overall percentage mortality by treatment arm is shown in Fig. 1. Because chlorfenapyr shows the property of delayed mortality, which reaches a zenith 72 h after mosquitoes enter into the huts with chlorfenapyr treated nets, both 24 h and 72 h mortality are presented in Table 2. Percentage mortality corrected for untreated net control is also shown.

**Table 2:** Percentage mortality of *Anopheles funestus* corrected for control mortality 24 h and 72 h after exposure during the two experimental huts trials

Trial	Effect	Holding period	Untreated net	Interceptor LN	Interceptor LN	Interceptor G2	Interceptor G2	Chlorfenapyr CTN
		Hours	0 washes	0 washes	20 washes	0 washes	20 washes	0 washes
1	Total number dead	24, 72	13, 29	14, 34	14, 33	30, 38	66, 92	23, 35
	% Mortality, overall	24	9^a1^ (4–19)	15^a1^ (8–27)	14^a1^ (8–24)	48^b1^ (36–59)	50^b1^ (41–58)	43^b1^ (30–56)
	% Mortality, control corrected	24	-	6^a1^ (0–19)	6^a1^ (0–17)	42^b1^ (30–54)	44^b1^ (35–54)	37^b1^ (30–52)
	% Mortality overall	72	21^a2^ (11–36)	37^c2^ (25–51)	34^c2^ (22–48)	60^b1^ (48–71)	70^d2^ (61–78)	65^bd2^ (48–78)
	% Mortality, control corrected	72	-	21^a2^ (5–33)	17^a2^ (2–34)	50^b1^ (34–62)	62^c2^ (51–72)	55^bc2^ (35–73)
2	Total number dead	24, 72	12, 14	10, 13	13, 14	31, 55	14, 42	20, 33
	% Mortality, overall	24	11^a1^ (5–17)	13^a1^ (5–20)	17^ac1^ (8–25)	30^b1^ (21–39)	16^ac1^ (9–24)	27^bc1^ (17–37)
	% Mortality, control corrected	24	-	2^a1^ (0–10)	7^a1^ (0–16)	22^b1^ (12–32)	6^a1^ (1–15)	18^b1^ (7–30)
	% Mortality overall	72	13^a1^ (7–19)	16^a1^ (8–24)	18^a1^ (9–27)	53^b2^ (44–63)	49^b2^ (38–59)	45^b2^ (33–55)
	% Mortality, control corrected	72	-	4^a1^ (0–13)	6^a1^ (0–16)	46^b2^ (35–58)	41^b2^ (29–53)	36^b2^ (23–48)
1	% Overall Killing Effect	24	-	5^a^	5^ab^	38^b^	41^c^	33^ab^
		72	-	17^b^	13 ^b^	30^a^	49^a^	44^a^
2	% Overall Killing Effect	24	-	0^a^	1^a^	17^a^	2^a^	7^a^
		72	-	0^a^	0^a^	38^b^	26^b^	18^b^

The numbers in the same row sharing the same letter superscript do not differ significantly (p > 0.05)

In the first trial, control-corrected mortality of *An. funestus* after 24 h was 5–6% in the huts with the unwashed IG1 and in the huts with the IG1 washed 20 times (Table 2). Mortality in these treatment arms was significantly different from the mortality in the huts with the unwashed IG2 (42%), the IG2 washed 20 times (44%) and the chlorfenapyr CTN (37%). After 72 h, control corrected mortality was significantly higher than after 24 h across most of these treatments (Table 2). Mortality was significantly higher in the huts with the IG2 unwashed and washed 20 times compared with the IG1 unwashed and washed 20 times treatments.

In the second trial, the trend was slightly different. Control corrected mortality significantly increased once again between 24 and 72 h with the unwashed IG2 (from 22 to 46%), the IG2 washed 20 times (from 6 to 41%) and the chlorfenapyr CTN (from 18 to 36%) (Table 2). But unlike the first trial, control-corrected mortality showed no significant change between 24 and 72 h with the unwashed IG1 (1.9% to 3.8%) and with the IG1washed 20 times (6.6% to 5.6%). Therefore, delayed mortality of *An. funestus* after 72 h was significantly pronounced only in the huts with the chlorfenapyr CTN, the unwashed Interceptor G2 and the Interceptor G2 washed 20 times.

Natural mortality of *An. funestus* after 72 h in the huts with the untreated nets in the first trial was significantly lower (21%) than the overall mortality in huts with the IG1 unwashed (37%) or IG1 washed 20 times (34%). In the second trial, natural mortality after 72 h in huts with the untreated nets was lower (13%) than in the first trial (21%), but on this occasion the untreated nets showed no difference in mortality compared to IG1 unwashed (16%) or IG1 washed 20 times (18%) which also stayed low. A further difference between the two trials: in the first, both IG2 and IG1 showed significantly delayed mortality between 24 and 72 h, in the second trial only IG2 showed significantly delayed mortality between 24 and 72 h and not IG1.

Because the untreated net control showed 21% (trial 1) and 13% (trial 2) mortality after the 72 h holding period, the observed mortality of IG2 presented in Fig. 1 after 72 h observation was considerably higher (range: 49–70%) than the control-corrected mortality (range: 41–62%) presented in Table 2.

The ‘overall killing effect’ by the IG1 and IG2 interventions were consistent with percentage mortality of the IG1 and IG2 treatments observed in the huts. In the first and second trials, IG1 killed up to 16% and 0% of *An. funestus,* respectively, and IG2 killed up to 49% and 38%, respectively.

### Meta-analysis of mortality

**Fig. 2:**
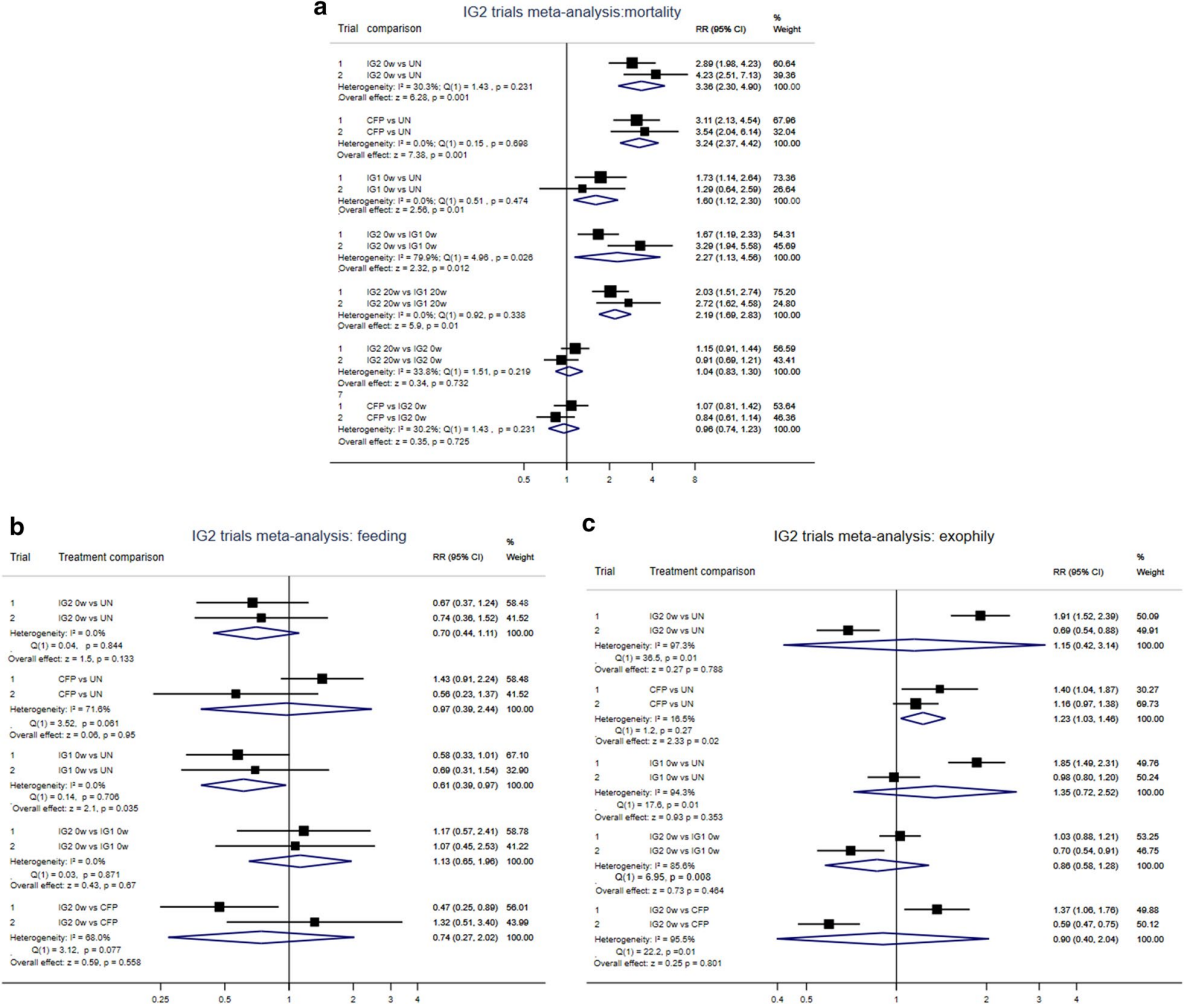
**a** Interceptor G2 versus Interceptor LN: meta-analysis of the two hut trials: mortality.** b** Interceptor G2 vs Interceptor LN Metanalysis of the two trials: feeding.** c** Interceptor G2 vs Interceptor LN Metanalysis of the two trials: exophily

In the meta-analyses of mortality between the two trials, the comparison of relative risk between the unwashed IG2 and the untreated net was 3.36 (CI 2.3, 4.9) (P = 0.001). The comparison of mortality relative risk between the chlorfenapyr CTN and untreated net, 3.24 (CI 2.4, 4.2) (P = 0.001) was, therefore, quite similar to that of the unwashed IG2 and untreated net. The comparison of relative risk between the unwashed IG1 and the untreated net was rather less (1.60, CI 1.1–2.3) (P = 0.01), indicating a smaller effect size of alpha-cypermethrin on mortality. The effect of the comparison between IG2 and IG1 was 2.27 (1.1, 4.6) (P = 0.012), confirming the greater contribution of chlorfenapyr than of alphacypermethrin to IG2 mortality. This was further confirmed by the comparison of chlorfenapyr CTN to IG2: the risk ratio was a non-significant 0.96 (0.7, 1.23) (P = 0.231) implying that chlorfenapyr was making most of the contribution to mortality in IG2 and not alpha-cypermethrin. The similarity of relative risk between unwashed IG2 and IG2 after 20 washes (1.04, CI 0.8–1.3) (P = 0.73) indicated no loss of mortality effect in IG2 between 0 and 20 washes (Fig. 2a).

### Blood feeding rates and personal protection

In the first trial, the percentage blood-feeding of *An. funestus* was significantly greater in the huts with the untreated net than in the huts with IG1 and IG2. There were no significant differences in blood-feeding rates between the huts with the IG1 or the IG2, with or without washing (Table 3). Neither was there significant difference in percentage blood-feeding between untreated net and chlorfenapyr CTN nor evidence of blood-feeding inhibition due to chlorfenapyr presence (percentage blood-feeding was greater in the huts with the chlorfenapyr CTN).

**Table 3:** Blood-feeding and blood-feeding inhibition of *Anopheles funestus* collected in the two experimental hut trials

Trial	Effect	Untreated net	Interceptor LN	Interceptor LN	Interceptor G2	Interceptor G2	Chlorfenapyr CTN
		0 washes	0 washes	20 washes	0 washes	20 washes	0 washes
1	Total number blood fed	36	14	16	11	23	20
	% Blood fed (95% C.I.)	26^ac^ (20–33)	15^b^ (7–28)	17^b^ (8–30)	18^bc^ (10–29)	17^b^ (10–29)	37^a^ (22–55)
	% Blood feeding inhibition	0	42	36	32	33	0
	% Personal protection	0^a^	61^bc^	56^bc^	69^b^	36^c^	44^bc^
2	Total number blood fed	16	8	7	11	13	6
	% Blood fed (95% C.I.)	14^a^ (8–24)	10^a^ (5–20)	9^a^ (4–21)	11^a^ (4–25)	15^a^ (7–30)	8^a^ (3–20)
	% Blood feeding inhibition	0	31	38	26	0	44
	% Personal protection	0^a^	50^a^	56^a^	31^a^	19^a^	63^a^

The numbers in the same row sharing the same letter superscript do not differ significantly (p > 0.05)

**Table 4:** Chemical analysis of insecticide on nets

Trial	Type of LLIN and wash treatment		Alpha-cypermethrin content (g/kg)				Chlorfenapyr content (g/kg)			
			Before washing*	After washing	Retention (%)	After trial	Before washing*	After washing	Retention (%)	After trial
1	Interceptor G1	0 wash	-	-	-	3.63	-	-	-	-
	Interceptor G1	20 washes	-	1.86	-	2.80	-	-	-	-
	Interceptor G2	0 wash	-	-	-	2.12	-	-	-	3.93
	Interceptor G2	20 washes	-	2.24	-	1.86	-	3.93	-	2.46
	Chlorfenapyr CTN	0 wash	-	-	-	-	-	-	-	6.29
2	Interceptor G1	0 wash	5.55	5.37	-	5.18	-	-	-	-
	Interceptor G1	20 washes	5.38	1.59	30%	1.52	-	-	-	-
	Interceptor G2	0 wash	2.81	2.77	-	2.85	5.22	5.12	-	5.08
	Interceptor G2	20 washes	2.79	1.65	59%	1.75	5.18	1.66	32%	1.79
	Chlorfenapyr CTN	0 wash	-	-	-	-	4.68	3.18	-	3.80

* No individual samples were cut from nets pre-washing in the first trial

In the second trial, while the percentage blood-feeding may have seemed greater in the huts with the untreated net than in the huts with the unwashed IG1 or IG1 washed 20 times the differences were not significant. Once again, no significant differences were evident between any of the IG1 and IG2 treatments. In the second trial, the difference between the untreated net and the chlorfenapyr CTN was also non-significant. Seven of the eight treatments that did show some degree of blood-feeding inhibition contained an alpha-cypermethrin component whether in IG1 or when twinned with chlorfenapyr in IG2.

In the first trial, personal protection in huts with IG1 and IG2 was significantly greater than in huts with the untreated nets. The chlorfenapyr net also showed significantly greater personal protection compared to untreated nets. In the second trial, while the numbers of *An. funestus* that were blood fed were also less in huts with the insecticide treated nets, neither the IG1, IG2 nor the chlorfenapyr treatments showed significant reduction in number blood-fed compared numbers blood-fed in huts with the untreated net. From these results it is not possible to conclude definitively that chlorfenapyr has no role in personal protection in huts with the chlorfenapyr treated net, but as regards personal protection in IG2, it would seem that that the alphacypermethrin component has the major role mediated through reduced blood-feeding just as in IG1.

### Meta-analysis of percentage blood feeding

In the meta-analyses of blood-feeding between the two trials, the comparison of relative risk between the unwashed IG2 *versus* the untreated net was 0.70 (CI 0.44, 1.11) (P = 0.133). The comparison of relative risk between the unwashed IG1 versus the untreated net was also quite similar (0.61, CI 0.39, 0.97) (P = 0.035) to that of IG2 above (Fig. 2b). The comparison of relative risk between the chlorfenapyr CTN versus the untreated net was 0.97 (CI 0.39–2.44) (P = 0.95). Considering these results in reverse order: chlorfenapyr treatment seems to have no effect on blood-feeding compared to no treatment. Alpha-cypermethrin was the sole AI contributing to reduced blood-feeding in the comparison of IG1 to untreated net. The inference is, the contributing active ingredient to reduced blood-feeding in IG2 versus untreated net is the alpha-cypermethrin rather than the chlorfenapyr. Further, the meta-analysis of relative risk of the comparison of IG2 versus chlorfenapyr CTN was 0.74 (0.3–2.0) (P = 0.67). This relative risk, being in the same direction as the relative risk between IG1 versus untreated net (0.61, 0.39–0.97) may support the interpretation that the chlorfenapyr has little or no role in blood-feeding in IG2 nor does it antagonize the positive effect alpha-cypermethrin has on reducing blood feeding in IG2 (Fig. 2b).

### Exiting rates

In the first trial, mosquito exiting rates were significantly higher in the huts with IG1, IG2 and chlorfenapyr CTN treatments compared to the huts with untreated nets (Table 1). In the second trial the exiting rates from huts with IG1, 1G2 and chlorfenapyr CTN were not significantly different from exiting rates from huts with the untreated net nor from one another (Table 1).

### Meta-analysis of enhanced exiting

In the meta-analysis these differences between the first and second trials led to heterogeneity in several of the comparisons of relative risk for exiting rates between treatments. No comparison between IG2 and any other treatment (untreated net, alpha-cypermethrin net, chlorfenapyr net) was significantly different from unity (Fig. 2c).

#### Anopheles gambiae sensu lato

Abundance of *An. gambiae* was very low in trial 1 with only 42 mosquitoes collected from the six treatments over 54 nights. However, differences in mortality were observed at 72 h with significantly higher mortality observed in huts with unwashed IG2 and IG2 washed 20 times (14/16) compared to IG1 (4/11) or untreated nets (1/10) (Supplementary file), which is consistent with the *An. funestus* dataset trends. Insufficient *An. gambiae* were collected during trial 2 for formal analysis.

### Chemical analysis

The mean alpha-cypermethrin content in unwashed IG2 for trial 2 (the WHO trial) was 2.81 g/kg (Table 4). The nets complied with the target dose of 2.4 g/kg ± 25% for 100 denier yarn. The mean chlorfenapyr content in unwashed IG2 for trial 2 was 5.22 g/kg. The nets complied with the target dose of 4.8 g/kg ± 25%. The within-net variation showed an acceptable homogeneity of active ingredient within the nets. After 20 washes the IG2 alpha-cypermethrin content for trial 2 was 1.65 g/kg, corresponding to an overall alpha-cypermethrin retention of 59%. The chlorfenapyr content was 1.66 g/kg after 20 washes, corresponding to an overall chlorfenapyr retention of 32% for trial 2. Netting samples were not kept back pre-washing in trial 1 for chemical analysis and therefore retention of chlorfenapyr and alpha-cypermethrin in IG2 after washing could not be accurately estimated. However, chemical analyses were conducted after the nets had been washed and tested in the huts and data was consistent with trial 2 post-trial retention estimates (see Table 4). The mean alpha-cypermethrin content in unwashed IG1from trial 2 was 5.55 g/kg. The alpha-cypermethrin content after twenty washes was 1.59 g/kg, corresponding to alpha-cypermethrin retention of 30% in IG1.

### Supporting bioassay tests on Interceptor and Interceptor G2 nets used in the hut trials

The purpose of the supplementary bioassays was to sample netting from the IG2 and IG1 used in the experimental hut trials to 1) test bio-efficacy against pyrethroid resistant (Zenet) and susceptible (Kisumu) strains in mosquito bioassay, 2) confirm the bioefficacy of alpha-cypermethrin and chlorfenapyr components after multiple washing, 3) examine the capacity of tunnel tests to predict the performance IG2 netting under simulated hut conditions to control *An gambiae s.s*.

**Fig. 3:**
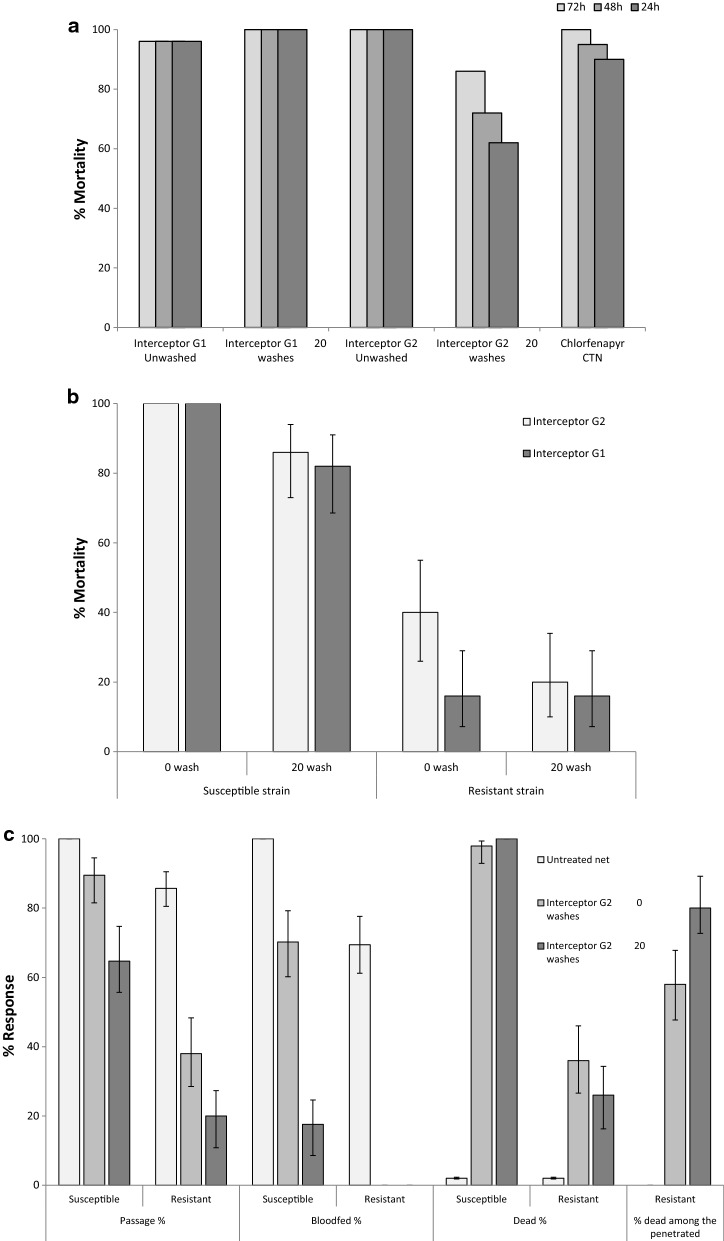
**a** Cone bioassay test on unwashed and 20 times washed Interceptor G2 and Interceptor LN nets using pyrethroid susceptible *Anopheles gambiae* Kisumu. **b** Cone bioassay test mortality on unwashed and 20 times washed Interceptor LN and Interceptor G2 nets using *Anopheles gambiae* pyrethroid susceptible Kisumu and pyrethroid resistant Zenet strain. Three-minute exposure and 72 h holding. **c** Tunnel tests with unwashed and washed Interceptor G2 netting against *Anopheles gambiae* pyrethroid susceptible (Kimumu) and pyrethroid resistant (Zenet) mosquitoes: % passage, % feeding, % mortality

Standard WHO Cone bioassay tests on nets with 3 min exposure of the susceptible strain and a 72 h holding period, induced mortality of 96% and 100% on the unwashed IG1 and the IG1 washed 20 times. With the chlorfenapyr CTN, mortality was 90%, 95% and 100% after 24 h, 48 h and 72 h. For the unwashed IG2, mortality was 100% after 24 h exposure. For IG2 washed 20 times mortality was 62%, 72% and 86% after 24 h, 48 h and 72 h intervals (Fig. 3a).

In further supplementary 3 min cone tests using the susceptible strain, mortality was 100% on unwashed IG1 and IG2, and on the IG1 and IG2 washed 20 times mortality was reduced to 82% and 86%, respectively. With the Zenet pyrethroid resistant strain, cone mortality was reduced to 16% and 40% with unwashed IG1 and IG2, respectively, and 16% and 20% respectively after 20 washes (Fig. 3b).

Supplementary tunnel tests were conducted using susceptible and resistant strains tested on unwashed IG2 and IG2 washed 20 times (Fig. 3c). With untreated netting, 100% of the susceptible and 86% of the resistant mosquitoes penetrated the holes into the baited chamber, 100% of the susceptible and 69% of the resistant mosquitoes blood-fed, and 2% of the susceptible and 2% of the resistant mosquitoes died. With unwashed IG2 netting fewer of the susceptible (89%) and resistant (38%) mosquitoes penetrated the holes, and even fewer susceptible (70%) and resistant (0%) mosquitoes’ blood-fed. However, 98% of the susceptible and 36% of the resistant mosquitoes were killed by the unwashed IG2 their attempts to feed. With the IG2 washed 20 times, a smaller percentage of the susceptible (65%) and resistant (20%) mosquitoes penetrated the holes (surprisingly), fewer susceptible (18%) and resistant (0%) blood-fed, and yet 100% of susceptible and 26% of resistant mosquitoes were killed by the IG2 washed 20 times. The newcomer Zenet strain was evidently less well adapted to the tunnel test, penetrating holed netting and responding/feeding on guinea pigs less well than did the long-established Kisumu.

### Comparison of supplementary bioassay tests with hut trial results

Comparing the laboratory cone and tunnel bioassay results against the pyrethroid resistant *An gambiae s.s.* strain and the experimental hut results against the wild pyrethroid resistant *An funestus* population, both types of bioassay predicted the response in the hut to the pyrethroid-only IG1: mortality was 16% in the cone and 13% in the hut against the unwashed IG1, and 16% in the cone and 11% in the hut against the IG1 20 times washed (averaged control-corrected mortality). When tested against the unwashed IG2, mortality was 40% in the cone, 36% in the tunnel and 51% in the hut; when tested with the 20 times washed IG2 mortality was 20% in the cone, 26% in the tunnel and 46% in the hut.

With the unwashed and washed IG2, percentage passage and percentage blood-feeding in the tunnel test were significantly lower with the newly-colonised resistant Zenet strain as compared to the long-established susceptible Kisumu strain. While up to 70% of Kisumu blood-fed after penetrating the IG2 netting, none (0%) of the Zenet strain blood-fed through IG2. And while high mortality of Kisumu (up to 70%) was recorded with IG2, low mortality was recorded against unwashed and washed IG2 (36% and 26%, respectively). This very much reflected the new adaptation of the Zenet strain to the tunnel test, possibly an avoidance or irritation of the treated net, or ‘reluctance’ to feed on guinea pigs. However, for those Zenet strain mosquitoes that did penetrate the netting, mortality inflicted by unwashed and 20 times washed IG2 was high, 58% and 80% respectively, and more closely resembled mortality in experimental huts.

This series of bioassay tests demonstrates that the chlorfenapyr component of IG2 LN makes the major contribution to controlling pyrethroid resistant *An. gambiae* and *An. funestus*. The tunnel tests were more predictive of efficacy in experimental huts whilst cone bioassays were less predictive.

## Discussion

Novel alternative insecticides which can complement the pyrethroids on LLIN and improve the control of pyrethroid resistant vectors are urgently needed to sustain progress against malaria. The objective of the present study was to determine the efficacy and wash-fastness of the chlorfenapyr-alphacypermethrin mixture net, Interceptor G2 LN, unwashed and after 20 washes, against the primary pyrethroid-resistant vectors *An. funestus* and *An. gambiae s.s.* under household-like conditions compared to the standard pyrethroid-only net Interceptor LN (IG1). Previously this very team had participated in the development and evaluation of IG1 against *An. funestus* and *An. gambiae s.s.* 10–14 years ago when these species were pyrethroid susceptible in NE Tanzania [8, 20]. Latterly this team`s participation was extended to development and evaluation of the new generation long-lasting net IG2 against the *An. gambiae* sibling species *An. coluzzii* in Benin, W Africa, and *An. arabiensis* in Kilimananjaro, Tanzania, where the species had become pyrethroid resistant [13–17]. Two trials were more recently extended to Muheza, NE Tanzania, aimed at evaluating IG2 against pyrethroid resistant *An. gambiae s.s.* and *An. funestus*. Only *An. funestus* was caught in significant numbers. In the meta-analysis of the two trials, the mortality induced by IG2 against *An. funestus* was 3.4 times higher than with untreated nets and 2.3 times higher than with IG1. The comparison of mosquito mortality between the unwashed IG2 and IG2 washed 20 times produced a relative risk of 1.04 (CI 0.83–1.30) indicating no loss of efficacy of IG2 over 20 washes. This means IG2 exceeds by a factor of 2.3 the mortality criterion required by WHO PQT to grant the product LLIN status [32]. The comparison of chlorfenapyr CTN with IG2 confirmed that the chlorfenapyr component of IG2 was the main contributor to mosquito mortality and net efficacy. However, it was also confirmed that the pyrethroid continues to have a valuable role with respect to blood-feeding inhibition, repellency and personal protection. The pyrethroid contributed 39% protection against blood-feeding of pyrethroid-resistant *An. funestus* in IG1 and 30% protection in IG2 compared to untreated nets. This was not far short of the 32% blood-feeding inhibition shown by IG1 against pyrethroid-susceptible *An. funestus* in Zenet hut trials over 10 years ago [20].

More important than the demonstration of equivalence of blood-feeding inhibition in resistant *An. funestus* was the clear demonstration of superior mortality of IG2 against resistant *An. funestus* that approached the mortality that IG1 once showed against *An. funestus* and *An. gambiae s.s.* in NE Tanzania before resistance evolved. In 2010, IG1 induced a control-corrected mortality of 80% among susceptible *An. funestus* when unwashed and 60% after 20 washes. In 2015–2016, IG1 only induced a control-corrected mortality of 12% among pyrethroid resistant *An. funestus* unwashed and of 11% after 20 washes. In contrast, IG2 induced a control-corrected mean mortality of 49% among pyrethroid resistant *An. funestus* when unwashed and 52% after 20 washes. While the performance of unwashed IG2 did fall a little short of the mortality induced by unwashed IG1 in 2006 against susceptible *An. funestus*, the mortality that the 20-times washed IG2 demonstrated against resistant *An. funestus* in 2016 was comparable to the efficacy 20-times washed IG1 demonstrated against susceptible *An. funestus* in 2006. Other recent experimental hut trials in West Africa in which IG2 has generated high mortality include *An. coluzzii* in Benin (71%, 65%), in Burkina Faso (76%, 75%) and Côte d’Ivoire (90%, 82%) when unwashed and washed 20 times, respectively. This is comparable mortality to that achieved with IG1 and other pyrethroid-only nets in the 1990s and new millennium when standard ITN and LLIN were first demonstrating malaria control and personal protection [37]. Considering the impact of ITN and LLIN then, it is reasonable to anticipate that IG2 and other Dual-AI will achieve comparative control of pyrethroid-resistant mosquitoes as standard LLIN once did against susceptible mosquitoes.

It is certainly the case that high intensity resistance means that standard LLIN are no longer preventing malaria as they once did. In countries and regions bordering Lake Victoria, for example, standard LLIN no longer appear to be reducing malaria despite maintenance of high coverage [9, 38, 39]. A cluster randomised trial of standard pyrethroid LLIN conducted in the region of high resistance, Kagera, on the western shore of Lake Victoria, Tanzania, could only demonstrate stasis in 2018 after introduction of new pyrethroid-only LLIN [9] but in adjacent clusters which were randomised to receive pyrethroid-PBO synergist LLIN there was a significant reduction in entomological inoculation rate and malaria prevalence [10].

The only putative insecticide mixture LLIN on the horizon, apart from the pyrethroid-chlorfenapyr net IG2, is a net treated with pyrethroid and pyriproxifen which is a mosquito sterilant and insect growth regulator. In a stepped wedge cluster randomised trial conducted in Burkina Faso a reduced malaria incidence rate of 12% was observed in the intervention arm compared to the control, a standard pyrethroid-only LLIN [40, 41]. As a mixture of two adulticides, IG2 would appear to hold more promise. Owing to the diversity of novel AI and modes of action being tested on LLINs, the WHO is no longer willing to accept entomological evidence as generated in experimental hut trials as adequate evidence for recommendation of a novel LLIN class. Since 2017, the WHO has required all new classes of LLIN to be subject to cluster randomized trials (CRT) with malaria control outcomes before they can gain approval or recommendation for wide-scale use as new methods of malaria control [42]. Chlorfenapyr is currently the only novel adulticide being evaluated on LLIN in a CRT. Such a trial takes at least 2 years to complete. This means that chlorfenapyr is a very precious AI, squandered at our peril. If chlorfenapyr fails due to evolution of resistance, there will be only PBO and piriproxyfen left in the armoury for use on nets. Fortunately, chlorfenapyr is novel chemistry and there is no sign of resistance so far, but resistance will evolve just as it always does. What must be done now is to identify ways preserve this AI as much as it is used to good effect. There is temptation to use it as an IRS insecticide too. In hut trials, it appears less effective applied as an IRS adulticide and the WHO proposes cluster randomized trial evidence of malaria effect [43, 44]. Blanket IRS coverage may accelerate resistance selection, as was demonstrated after 7 years of pyrethroid IRS in Kagera region that led to premature loss of pyrethroid effectiveness in LLIN just as LLIN were being scaled up [9, 10]. What is needed is a far-sighted resistance management strategy which prioritizes PBO, chlorfenapyr, and the few AI that can be used safely on nets and reduces their use in other applications, like IRS, if there are good alternatives that can be used or rotated to reduce selection pressure on chlorfenapyr in IG2.

## Conclusion

Novel alternative insecticides suitable that can complement the pyrethroids and improve the control of pyrethroid resistant malaria vectors are urgently required for sustaining LLIN as a means of malaria control. The mortality of pyrethroid resistant *An. funestus* induced by unwashed and 20 times washed Interceptor G2 appears to meet the entomological requirements set by the WHO for efficacy and wash-resistance. Thus far there is no epidemiological evidence to back up the entomological evidence nor any knowledge of how long Interceptor G2 LN or its chlorfenapyr component will remain effective under field conditions. Therefore, large-scale cluster randomized trials of Interceptor G2 with epidemiological end-points are an essential next step. A CRT in NW Tanzania against *An. funestus* and *An. gambiae* is due to report in mid-2021 for recommendation as a new class of LLIN product to WHO.

## Data Availability

The datasets used and/or analysed during the current study are available from the corresponding author on reasonable request.
